# Analysis of Breast Cancer Screening Results and Influencing Factors of Breast Cancer in Guangdong Province from 2017 to 2021

**DOI:** 10.1007/s44197-023-00176-3

**Published:** 2024-01-15

**Authors:** Li Wu, Guo-Zhen Chen, Zu-Rui Zeng, Cun-Wei Ji, An-Qin Zhang, Jian-Hong Xia, Guo-Cheng Liu

**Affiliations:** 1 Guangdong Women and Children Hospital, Xingnan Road 521, Guangzhou, 511442 Guangdong China; 2 School of Basic Medicine and Public Health, Jinan University, Guangzhou, 510632 Guangdong China

**Keywords:** Breast neoplasms, Breast cancer screening, Epidemiology, Influencing factors

## Abstract

**Backgrounds:**

Breast cancer screening plays an important role in the early detection, diagnosis and treatment of breast cancer. The aim of this study was to evaluate the screening results and explore the influencing factors of breast cancer detection rate in Guangdong.

**Methods:**

This cross-sectional study was conducted among 2,024,960 women aged 35–64 in Guangdong Province during 2017–2021. The data about breast cancer screening information were collected from the Guangdong maternal and child health information system. Descriptive statistical analysis was used to explain demographic characteristics and results of breast cancer screening. The generalized linear regression model was applied to analyze the related influencing factors of breast cancer detection rate.

**Results:**

The estimated detection rate of breast cancer in Guangdong Province is 70.32/10^5^, with an early diagnosis rate of 82.06%. After adjusting covariates, those women with older age (45–55 [OR (95% CI) 2.174 (1.872, 2.526)], 55–65 [OR (95% CI) 2.162 (1.760, 2.657)]), education for high school ([OR (95% CI) 1.491 (1.254, 1.773)]) and older age at first birth ([OR (95% CI) 1.632 (1.445, 1.844)]) were more likely to have higher detection rate of breast cancer. No history of surgery or biopsy ([OR (95% CI) 0.527 (0.387, 0.718)]), no history of breast cancer check ([OR (95% CI) 0.873 (0.774, 0.985)]) and no family history of breast cancer ([OR (95% CI) 0.255 (0.151, 0.432)]) women were more likely to screen negative for breast cancer (*P* < 0.05).

**Conclusion:**

The detection rate of breast cancer in screening showed an increasing trend year by year in Guangdong Province. Older age, education for high school and older age at first birth were risk factors for breast cancer detection rate, while no surgery or biopsy history, no family history of breast cancer and no history of breast cancer check were protective factors.

## Introduction

Breast cancer is the most common malignancy among women in the world. The American Cancer Society in 2022 depicts that for women, breast cancer in the United States alone accounts for 31% of all new cancer diagnoses[1], as well as in Korea[2]. The incidence of breast cancer ranked first in female cancers in China, and there was a dramatic increase in incidence and mortality rates[3]. Breast cancer screening, an important method for secondary prevention, is considered as an effective method to reduce breast cancer mortality and incidence rate, and it can also improve the survival rate and prognosis of women with breast cancer[4]. The Breast Screening Australia Program showed that screening was associated with between a 30%–41% reduction in breast cancer mortality[5].

In response to the government's call, Guangdong Province launched a free breast cancer screening program for rural women in 2009. In 2020, the screening target expanded from rural women to urban women. Since the inception of the program, the breast cancer screening model has been continuously optimized. At present, the benefits of breast cancer screening have not yet been observed, but the effects may manifest in the future[6]. Besides, the pathogenesis of breast cancer is complex, and breast disease is a multifactorial disease. Previous studies have shown that the main risk factors for breast cancer include older age, high body mass index or obesity, exposure to tobacco and alcohol and so on[7–12]. The risk factors of breast cancer among women in China are different from those in the West, and women in China have unique risk factors for breast cancer[13]. Therefore, it is necessary to further explore the influencing factors of breast cancer detection rate in China, and formulate a screening program suitable for women in China [14].

In this study, we analyze the breast cancer screening results and influence factors associated with breast cancer detection rate in Guangdong Province from 2017 to 2021. It can also provide a scientific basis for regulating screening strategies suitable for women and promoting breast cancer screening in China.

## Materials and Methods

### Subjects and Study Design

This cross-sectional study was conducted among 2,024,960 women covered by the Guangdong Province during 2017–2021. This study was based on breast cancer screening program in Guangdong province. The inclusion criteria were as follows:

1. aged 35–64 years.
2. voluntarily amenable to undergoing breast screening.

The exclusion criteria were as follows:

1. a history of breast cancer.
2. pregnant at the time of enrollment.
3. difficulty in obtaining information.

All the participants provided written informed consent for participation in the study. All study protocols were approved by the Ethics Committee of the Guangdong Women and Children Hospital.

### Data Collection

**Fig. 1:**
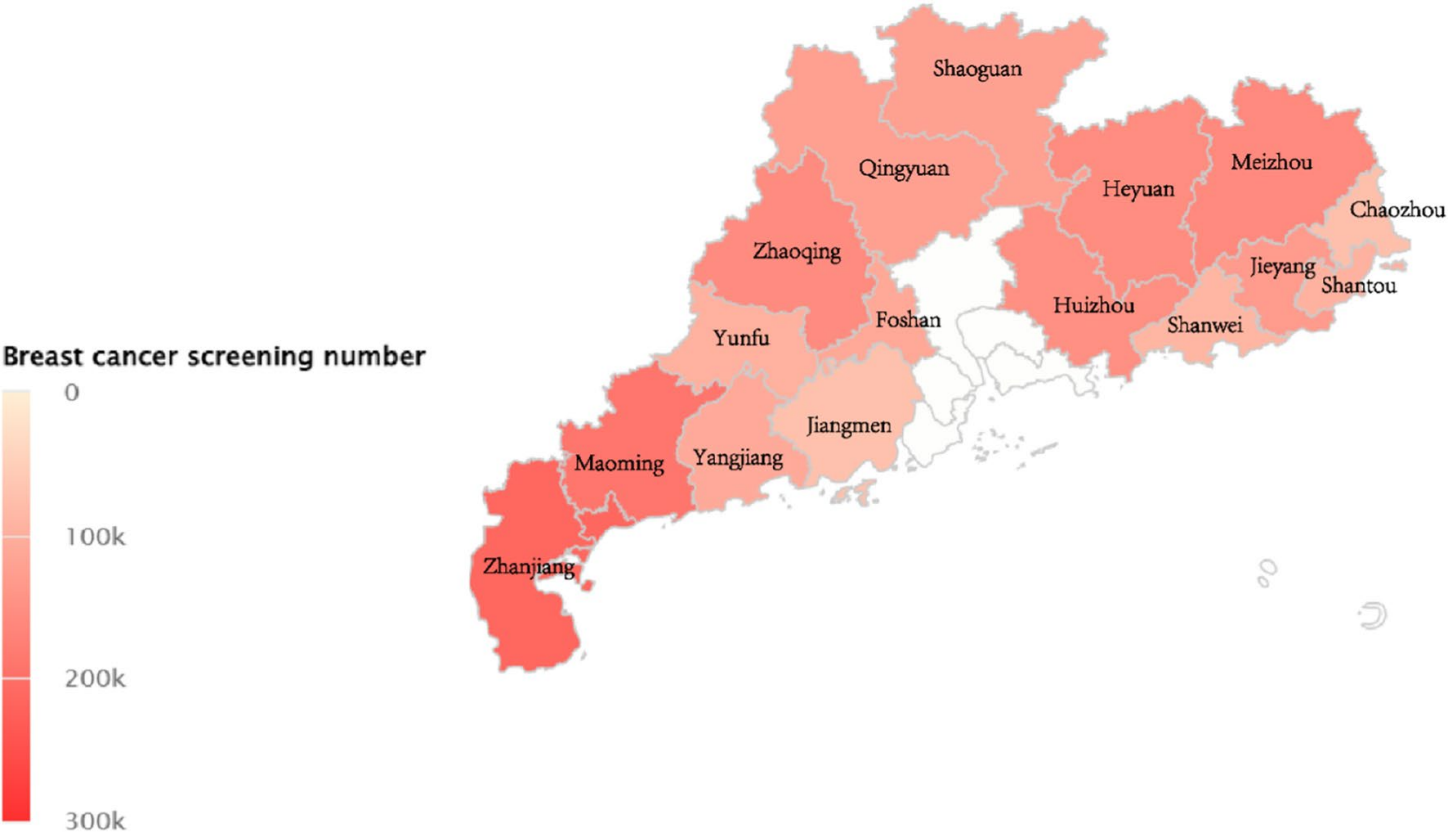
Geographical pattern of breast cancer screening population in different cities of Guangdong Province during 2017–2021

Firstly, the doctors conducted publicity and education on the screening subjects. The information includes basic and clinical information, results of breast examinations, and TNM stages and grades. As the largest province in China, Guangdong has a population of 126.84 million people and covers 17.97 km^2^ in the southernmost part of China, including 44 counties. The geographical distribution of screening populations is shown in Fig. 1.

### Study Methods

#### Screening Protocols and Procedures

The screening process included firstly carrying out breast cancer screening education activities for women of appropriate age through community, village doctors or other means. The dedicated person is responsible for conducting a questionnaire survey and provides breast cancer screening.

Question survey: face-to-face inquiry was used to investigate the basic information of the screening subjects, including age, education level, race, menstrual history, family history, birth history and so on.

Breast clinical examination and breast ultrasound examination: all women who participated in the screening underwent breast clinical examination and breast ultrasound examination. The results of breast color ultrasound examination were evaluated by Breast Imaging Reporting and Data System (BI-RADS).

Breast X-ray examination: those who were classified into Class 0 or Class 3 by BI-RADS in color ultrasound examination of the breast were subjected to breast X-ray examination, and the breast X-ray examination results were evaluated and reported by BI-RADS classification system.

**Fig. 2:**
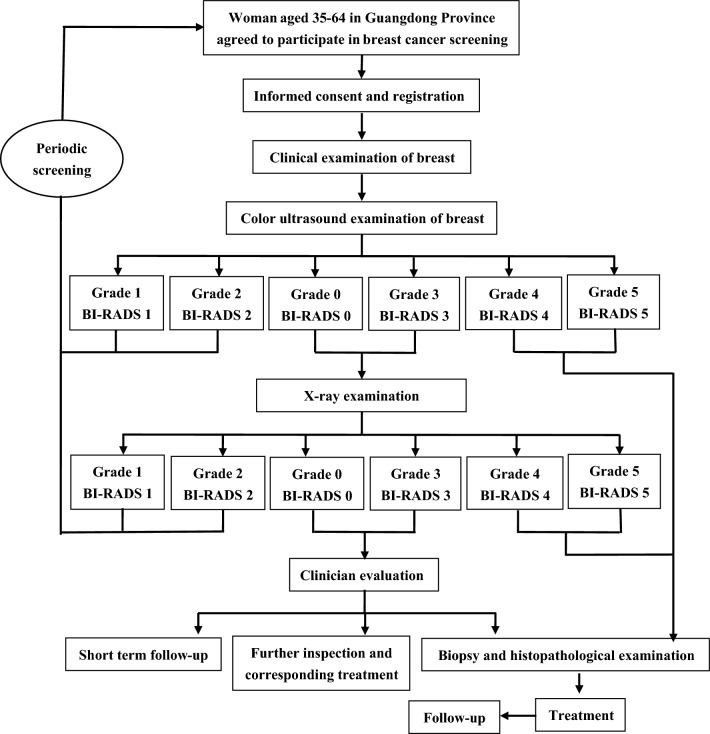
Breast cancer screening process in Guangdong province

Histopathological examination: if the BI-RADS classification result of color ultrasound examination of the breast is category 4 or 5, or the BI-RADS classification result of x-ray examination is category 4 or 5, a histopathological examination (biopsy) will be conducted directly. If the BI-RADS classification result of the breast X-ray examination is 0 or 3, the short-term follow-up, biopsy, histopathological examination or other examinations will be conducted after comprehensive evaluation by specialist above subtropical high level. A schematic of the screening process is shown in Fig. 2.

#### Relevant Definitions and Judgment Criteria

BI-RADS stands for Breast Imaging-Reporting and Data System which is a tool for qualitative expression and risk assessment in mammography and breast sonography. According to the diagnostic criteria of BI-RADS of American Radiological Society [15] all participants were divided into the following categories based on the BI-RADS results: a score of zero means insufficient mammography, a score of 1 no problem or lesion, score 2 benign findings, score 3 probably benign, score 4 suspected of malignancy, score 5 clear evidences of malignancy.

In this study, BI-RADS grade 1 and 2 were negative for breast cancer, BI-RADS grade 0 and 3 were suspiciously positive for breast cancer, and BI-RADS grade 4 and 5 were positive for breast cancer. The lesion confirmed by biopsy was breast cancer. Positive cases of breast cancer refer to breast precancerous lesions, breast ductal carcinoma in situ and breast invasive carcinoma confirmed by histopathological examination.

### Data Quality Control

The implementation of the screening agency inspectors must qualify, after a unified national special technical training, and training examination. A unified examination process, questionnaire design and reporting methods were adopted during the project implementation. Investigators should receive unified training and finally adopt strict and unified quality control measures. After the investigation information was collected, the data should be confirmed as complete and correct by the quality controller before entering into the database to ensure data quality. Every year, several counties are selected for on-site supervision, review of color ultrasound and breast X-ray imaging data, consultation of difficult cases and timely notification of supervision results. After the completion of screening, the experts in the project group shall 100% reread the uploaded images of breast cancer-positive cases and 1% conduct random inspection for negative cases.

### Statistical Methods

R 4.2.1 and Stata 16.0 were used for statistical analysis. Due to the missing ratio of educational level more than 20%, we applied the multiple imputation to interpolate missing values. Descriptive statistical analyses were applied to explain the demographic characteristics and the result of breast cancer screening programs during 2017–2021. Categorical variables were displayed as numbers (%). We described the year-to-year changes in the positive rates of different breast cancer screening methods and the overall breast cancer detection rates, and included the median of the annual detection rates as a continuous variable in the model for trend tests. At the same time, we calculated the rate of early diagnosis of breast cancer each year based on the screening population. We also standardized detection rates based on 2019 census data. Finally, we applied the Generalized linear regression model to analyze the related influencing factors of breast cancer detection. In addition, we created a heatmap to describe the screening population of each city in Guangdong Province. The age trend of breast cancer detection rate was displayed using a line chart. *P* < 0.05 was considered statistically significant.

## Results

### Population Characteristics

Table 1 presents all participants’ general characteristics (*n* = 2,024,960). The Han nationality of participants accounts for 99.19%, and the education background was mainly middle high school or below (79.47%). The majority of participants were 45 to 55 years old (40.68%). The vast majority of participants had menarche at least 12 years of age (99.32%), and nearly 40% had gone through menopause (38.01%). Few women give birth for the first time younger than 28 (82.74%), and the vast majority of women have a history of breastfeeding (95.52%). Most participants had no history of breast cancer check (75.91%) and surgery/biopsy (98.60%), and had no family history of breast (99.75%) or ovarian cancer (99.92%).

**Table 1:** The characteristics of participants in Guangdong Province from 2017 to 2021

Characteristics	Population (*n* = 2,024,960)	Proportion (%)
*Age (years)*		
35–44	623,448	30.79
45–54	823,758	40.68
55–64	577,754	28.53
*Ethnicity*		
Han	2,008,507	99.19
Non-Han	16,453	0.81
*Education*		
Primary school or below	799,862	39.50
Middle high school	809,341	39.97
High school	206,860	10.22
College or above	208,897	10.32
*Age at menarche (years)*		
> 12	13,774	0.68
≥ 12	2,011,186	99.32
*Menopause*		
Yes	732,798	36.19
No	1,292,162	63.81
*First birth*		
Yes	2,024,535	99.98
No	425	0.02
*Age at first birth (years)*		
< 28	1,675,516	82.74
≥ 28	349,444	17.26
*Breastfeeding history*		
Yes	1,934,156	95.52
No	90,804	4.48
*History of breast cancer check*		
Yes	487,748	24.09
No	1,537,212	75.91
*Surgery/biopsy history*		
Yes	28,419	1.40
No	1,996,541	98.60
*Family history of breast cancer*		
Yes	5062	0.25
No	2,019,898	99.75
*Family history of ovarian cancer*		
Yes	1611	0.08
No	2,023,349	99.92

### Breast Cancer Screening Results

Breast cancer screening includes clinical examination, color ultrasound, X-ray and pathological examination. Table 2 shows the conditions of these four breast cancer screening programs. The detection rate of each examination showed an increasing trend year by year (*P* < 0.001).

**Table 2:** The breast cancer screening methods

Year	Clinical examination		Color ultrasound			X-ray examination			Pathological examination	
	*N*	Suspicious malignancy (%)	*N*	Suspicious positive (%)	Positive (%)	*N*	Suspicious positive (%)	Positive (%)	*N*	Positive (%)
2017	78,379	25 (0.03)	78,237	2693 (3.44)	311 (11.55)	1641	510 (31.08)	102 (20.00)	587	23 (3.92)
2018	417,332	415 (0.10)	417,045	19,111 (4.58)	2731 (14.29)	12,529	4215 (33.64)	816 (19.36)	2356	196 (8.32)
2019	526,550	592 (0.11)	525,566	29,642 (5.64)	4339 (14.64)	19,998	7201 (36.01)	1243 (17.26)	8501	336 (3.95)
2020	485,556	630 (0.13)	485,283	37,920 (7.81)	5314 (14.01)	25,509	10,895 (42.71)	1466 (13.46)	2573	381 (14.81)
2021	517,143	555 (0.11)	517,008	42,861 (8.29)	6315 (14.73)	30,471	13,539 (44.43)	1750 (12.93)	3142	488 (15.53)
Total	2,024,960	2217 (0.11)	2,023,139	132,227 (6.54)	19,010 (14.38)	90,148	36,360 (40.33)	5377 (14.79)	17,159	1424 (8.30)
*P* value for trend		< 0.001***		< 0.001***	< 0.001***		< 0.001***	0.001**		< 0.001***

*P < 0.05; **P < 0.01; ***P < 0.001

**Fig. 3:**
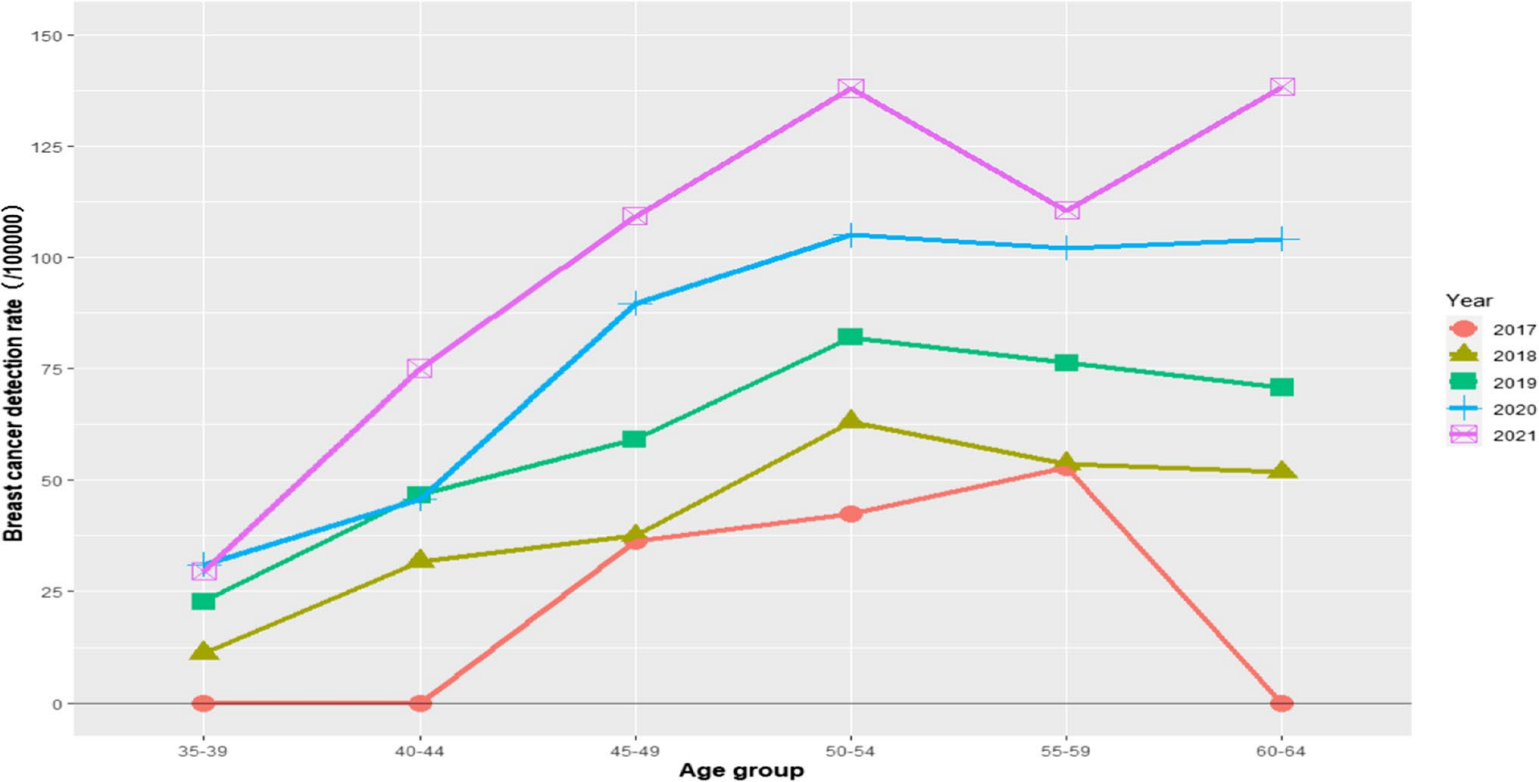
Age trend of breast cancer detection rate among 2017–2021

Table 3 summarizes the results of breast cancer screening in recent years. Between 2017 and 2021, 2,024,960 women were screened for breast cancer in Guangdong Province and 1424 were diagnosed as breast cancer (70.32/10^5^). Age-standardized detection rate was 30.85/10^5^. There were 462 women who had been diagnosed at an early stage, with an early diagnosis rate of 82.06%. breast cancer detection rate, national standard rate, and the number of early diagnoses have linear trends (*P* < 0.05). The change of breast cancer detection rate with age in different years is shown in the Fig. 3. At almost all age groups, breast cancer detection rates increased from 2017 to 2021.

**Table 3:** The detection of breast cancer

Year	Detection			Early detection	
	The number of breast cancer screening	Breast cancer number (rate/10^5^)	Age-standardized rate (1/10^5^)	The number of detection	The number of early detection (%)
2017	78,379	23 (29.34)	10.88	6	5 (83.33)
2018	417,332	196 (46.97)	19.62	51	40 (78.43)
2019	526,550	336 (63.81)	27.40	86	75 (87.21)
2020	485,556	381 (78.47)	35.61	180	139 (77.22)
2021	517,143	488 (94.36)	44.14	240	203 (84.58)
Total	2,024,960	1424 (70.32)	30.85	563	462 (82.06)
*P*-value for linear trend	0.106	< 0.001*	< 0.001*	0.002**	0.903

*P < 0.05; **P < 0.01; ***P < 0.001

### Influencing Factors of Breast Cancer Detection Rate

Adherence concerning the influencing factors was explored using a Generalized linear regression model. Age, education, age at first birth, history of breast cancer check, surgery or biopsy history and family history of breast cancer were associated with breast cancer-positive results after adjusting covariates (*P* < 0.05) (Table 4). Strong positively associations were found in the age of 44–55 [OR (95% CI) 2.174 (1.872, 2.526)] and 55–64 [OR (95% CI) 2.162 (1.760, 2.657)] years old, and in the first birth age for ≥ 28 years old [OR (95% CI) 1.632 (1.445, 1.844)]. Education for high school [OR (95% CI) 1.491 (1.254, 1.773)] was significantly positively associated with the breast cancer positive results after adjusting covariates. Compared with women who underwent breast cancer screening, women who had not been checked for breast cancer [OR (95% CI) 0.873 (0.774, 0.985)] were more likely to have a negative breast cancer test. Compared to women with a history of surgery or biopsy, women without a history of surgery or biopsy are more likely to screen negative for breast cancer [OR (95% CI) 0.527 (0.387, 0.718)]. Compared to women with a family history of breast cancer, women without a family history of breast cancer are more likely to screen negative for breast cancer [OR (95% CI) 0.255 (0.151, 0.432)].

**Table 4:** Univariate and multivariate analysis of breast cancer in Guangdong Province from 2017 to 2021

Variables	Number of persons checked	Detection rate (1/10^–5^)	Model 1 OR (95% CI)	*P*-value	Model 2 OR (95% CI)	*P-*value
*Age (years)*						
35–44	623,448	41.38	Ref		Ref	
45–54	823,758	84.86	2.051 (1.778–2.366)	< 0.001***	2.174 (1.872–2.526)	< 0.001***
55–64	577,754	80.83	1.954 (1.678–2.275)	< 0.001***	2.162 (1.760–2.657)	0.001**
*P*-trend				< 0.001***		< 0.001***
*Ethnicity*						
Han	2,008,507	70.15	Ref		Ref	
Non-Han	16,453	91.17	1.300 (0.781–2.162)	0.313	1.540 (0.925–2.564)	0.097
*Education*						
Primary school and below	799,862	66.89	Ref		Ref	
Middle high school	809,341	69.69	1.042 (0.926–1.173)	0.497	1.165 (1.031–1.315)	0.014
High school	206,860	88.47	1.323 (1.118–1.565)	0.001**	1.491 (1.254–1.773)	< 0.001***
College or above	208,897	67.98	1.016 (0.845–1.222)	0.864	1.200 (0.986–1.460)	0.068
*Age at menarche (years)*						
< 12	13,774	50.82	Ref		Ref	
≥ 12	2,011,186	70.46	1.387 (0.66–2.914)	0.389	1.362 (0.647–2.867)	0.415
*Menopause*						
No	1,292,162	63.23	Ref		Ref	
Yes	732,798	82.83	1.310 (1.180–1.455)	< 0.001***	1.074 (0.926–1.244)	0.346
*Age at first birth (years)*						
< 28	1,675,516	63.68	Ref		Ref	
≥ 28	349,444	102.16	1.605 (1.424–1.809)	< 0.001***	1.632 (1.445–1.844)	< 0.001***
*Breastfeeding history*						
Yes	1,934,156	70.42	Ref		Ref	
No	90,804	68.28	0.970 (0.752–1.251)	0.812	0.97 (0.751–1.253)	0.816
*History of breast cancer check*						
Yes	487,748	82.62	Ref		Ref	
No	1,537,212	66.42	0.804 (0.716–0.902)	< 0.001***	0.873 (0.774–0.985)	0.028*
*Surgery/biopsy history*						
Yes	28,419	158.34	Ref		Ref	
No	1,996,541	69.07	0.436 (0.324–0.586)	< 0.001***	0.527 (0.387–0.718)	< 0.001***
*Family history of breast cancer*						
Yes	5062	296.33	Ref		Ref	
No	2,019,898	69.76	0.235 (0.141–0.391)	< 0.001***	0.255 (0.151–0.432)	< 0.001***
*Family history of ovarian cancer*						
Yes	1611	62.07	Ref		Ref	
No	2,023,349	70.33	1.133 (0.159–8.054)	0.901	3.222 (0.434–23.906)	0.253

OR, odd ratio; *P*-t, *P* value for trend; CI, confidence interval; Model 1: unadjusted model, Model 2: adjusted for all covariates: age, nationality, education level, age at menarche, menopause, age at first birth, breastfeeding history, breast cancer examination history, surgery/biopsy history, family history of breast cancer and ovarian cancer

*P < 0.05; **P < 0.01; ***P < 0.001

## Discussion

The incidence and mortality of breast cancer, especially in China, increased rapidly in recent years, and breast cancer has become the most common malignant tumor in women[16]. The main aim is to detect and modify controllable carcinogenic factors, strive for early detection of malignancy, and thus reduce patient mortality[17]. The purpose of this study was to analyze the results of breast cancer screening and the influencing factors of breast cancer in Guangdong Province.

### Detection Results of Breast Cancer Screening

Between 2017 and 2021, the main methods of breast cancer screening in Guangdong include clinical examination, mammography, ultrasonography and histopathology.

In our study, the detection rate of suspicious malignancy in breast clinical examination significantly was increased in 2020. It is worth noting that many factors affect the sensitivity of breast clinical examination, such as the experience of clinicians, women's age, body mass index [18]. The suspicious positive detection rate of ultrasound examination and X-ray increased consistently, and the positive detection rate of X-ray decreased year by year. Education levels, being older and having a friend who had been diagnosed with breast cancer were also shown to be positively associated with the participation in either mammography or ultrasonography [19]. A report from IARC showed that screening women aged 50–69 years with mammography is associated with a 25% reduction in breast cancer mortality [20]. Although there is extensive controversy over the pros and cons of X-ray screening, the benefits outweigh the harms of mammography, and the efficacy achieved by screening with mammography is clear [21]. Considering the accessibility and relatively low cost of ultrasound examination in clinical practice in China, the guidelines suggest that mammography and ultrasound examination should be combined to carry out screening[22]. The pathological examination detection rate of breast cancer among women in Guangdong Province has exceeded 15% since 2020, with a significant increase. Histopathology and other diagnostic methods can confirm or exclude the diagnosis of breast cancer [23]. The economy can affect the distribution and availability of health resources, thus affecting the results of breast pathological examination[24]. In addition, the ultrasound examination would increase patient's recall rate and opportunities for breast pathology examination [25]. These screening methods complement each other.

The breast cancer detection rate reached 70.32/10^5^ and increased at almost all age groups by years in these 5 years. The incidence of breast cancer has increased slowly and the distribution of risk factors has been changing [26]. Breast cancer screening coverage in Guangdong Province in 2020 was expanded to include both urban and rural women. The sudden increase in the number of women being screened for breast cancer is likely to have an impact on the increasing trend of detection rate of breast cancer. The rate of early detection of breast cancer in 5 years did not change significantly.

### Influencing Factors of Breast Cancer Detection Rate

At present, researchers in China have been using the collected information of various risk factors to explore breast cancer risk models[27–29]. The influencing factors of breast cancer detection rate are still unclear. It is necessary to analyze the influencing factors of breast cancer detection rate using large sample screening data, so as to promote the establishment of standardized breast cancer screening and early diagnosis system in China.

Age is a risk factor for breast cancer, and women aged 45–54 years have the greatest risk of breast cancer comparing to other groups in this study. Women between 45 and 54 years old are in perimenopausal period, and the risk of breast cancer with abnormal estrogen level is higher. After the peak age, the incidence of breast cancer in Asian women began to decline gradually, which is consistent with this study[25]. In addition to 45–54 years, there is another age peak for breast cancer onset in China at 70–74 years[30]. Most breast cancer screening guidelines recommend mammographic screening for average risk individuals aged 40–74[31]. The main difference between the guidelines is whether to screen women aged 40–49[32]. The age of onset of breast cancer in China is much younger, with a median age of 50 years, and breast cancer is also the most common cause of death in women younger than 45 years[33].

As for the higher education level, we found it increases the detection rate of breast cancer. In agreement with our result, some studies have shown that the level of education is related to health beliefs, and their health beliefs affected breast cancer screening behaviors[34]. The higher the education level, the higher the health awareness of women and the more active they will take part in breast cancer screening[35]. Some researchers hold different views, they believe that women who have attained tertiary education might be overwhelmed with work schedules and not able to attend or book an appointment for breast cancer screening[36].

The risk of detecting breast cancer in women who are ≥ 28 years old for the first birth is 1.632 times that of women < 28 years old in our study. This increase is due to the late differentiation of breast cells after the first pregnancy, thus increasing the duration of the cancer susceptibility phase. Getting pregnant for the first time before the age of 30 should help reduce your risk of cancer[37]. Previous studies have shown that among menopausal or near-menopausal women, the cumulative incidence of breast cancer among women who gave birth for the first time at the age of 20, 25 and 35 (until the age of 70) decreased by 20%, 10% and increased by 5%, respectively, compared with those who had not given birth[38]. In addition, the risk of breast cancer in nulliparous women is 1.32 times that of multiparous women[39].

The results of our study showed that no history of cancer check was a protective factor for breast cancer detection. Women who have participated in breast cancer check may have high risk factors for breast cancer, and their awareness of health care is relatively strong, which increases the detection of breast cancer screening. A study, which provided the largest nationwide and population-based self-reported history of breast cancer screening in China in 2015, showed that nearly one-fifth of Chinese women ever had breast cancer screening[40].

In our research results, having a history of breast cancer surgery/biopsy history is a risk factor for breast cancer detection. Current guidelines in China indicate that a history of breast biopsy or surgery for a benign breast disease, or a history of pathologically proven atypical hyperplasia of the breast (lobular or ductal) can be defined as one of the conditions for a high-risk population[41]. For women with these above conditions, annual MAM or annual MRI was mainly recommended after diagnosis onward[42].

Family history of breast cancer is an unmodifiable risk factor for breast cancer and have been proposed as criteria for risk stratification[43]. Relevant studies have shown that women with a previous benign breast disease or a family history of breast cancer had higher cumulative risks of screening outcomes than women without these characteristics [44]. Women exposed to family history of breast cancer can adopt many healthy behavior changes to reduce the risk of breast cancer[45]. For example, women should be encouraged to be aware of and to discuss with a clinician. China Breast Surgery Society suggested that women who have the possibility of breast cancer genetic predisposition based on personal or family history should receive genetic counseling[46].

## Limitations

Our study had some limitations. First of all, the results of this study cannot be generalized to more developed areas due to the regional particularity of breast cancer screening program. As this analysis is based on a government-led public health service project, there are certain practical difficulties in the implementation process, resulting in the loss of follow-up in all aspects of screening. In addition, our findings cannot determine any causal inference about the relationship between the factors and the detection of breast cancer due to its non-experimental study design. There may be many factors not included in the study, such as screening satisfaction, economic income, body mass index and other important risk factors, which will weaken our final research results. Despite these limitations, this study can provide information that may be useful for both researchers and policy makers involved in public health programs.

## Conclusions

In summary, the breast cancer prevalence in Guangdong Province has reached 71.02/10^5^, and it has been increasing from 2017 to 2021. Older age, non-Han, education for high school, older age at first birth, menopause, surgery or biopsy history, family history of breast cancer and never participation in breast cancer screening before are dangerous factors for breast cancer. These findings will provide a scientific basis for China to specify scientific guidelines for breast cancer screening and explore intervention strategies to prevent breast cancer, so that more women can benefit from it.

## Data Availability

The datasets generated or analyzed during this study are available from the corresponding author on reasonable request.

## Abbreviations

TNM: Tumor node metastasis
BI-RADS: Breast imaging reporting and data system
CI: Confidence interval
